# Semantic segmentation of human oocyte images using deep neural networks

**DOI:** 10.1186/s12938-021-00864-w

**Published:** 2021-04-23

**Authors:** Anna Targosz, Piotr Przystałka, Ryszard Wiaderkiewicz, Grzegorz Mrugacz

**Affiliations:** 1 Department of Histology and Embryology, Medical University of Silesia, Faculty of Medical Sciences, 18 Medyków St., 40-752 Katowice, Poland; 2 Center for Reproductive Medicine Bocian, 26 Akademicka St., 15-267 Białystok, Poland; 3 Department of Fundamentals of Machinery Design, Silesian University of Technology, Faculty of Mechanical Engineering, 18a Konarskiego St., 44-100 Gliwice, Poland

**Keywords:** IVF, Human oocyte, Semantic segmentation, Image analysis, Artificial intelligence, Deep neural networks

## Abstract

**Background:**

Infertility is a significant problem of humanity. In vitro fertilisation is one of the most effective and frequently applied ART methods. The effectiveness IVF depends on the assessment and selection of gametes and embryo with the highest developmental potential. The subjective nature of morphological assessment of oocytes and embryos is still one of the main reasons for seeking effective and objective methods for assessing quality in automatic manner. The most promising methods to automatic classification of oocytes and embryos are based on image analysis aided by machine learning techniques. The special attention is paid on deep neural networks that can be used as classifiers solving the problem of automatic assessment of the oocytes/embryos.

**Methods:**

This paper deals with semantic segmentation of human oocyte images using deep neural networks in order to develop new version of the predefined neural networks. Deep semantic oocyte segmentation networks can be seen as medically oriented predefined networks understanding the content of the image. The research presented in the paper is focused on the performance comparison of different types of convolutional neural networks for semantic oocyte segmentation. In the case study, the merits and limitations of the selected deep neural networks are analysed.

**Results:**

71 deep neural models were analysed. The best score was obtained for one of the variants of DeepLab-v3-ResNet-18 model, when the training accuracy (Acc) reached about 85% for training patterns and 79% for validation ones. The weighted intersection over union (wIoU) and global accuracy (gAcc) for test patterns were calculated, as well. The obtained values of these quality measures were 0,897 and 0.93, respectively.

**Conclusion:**

The obtained results prove that the proposed approach can be applied to create deep neural models for semantic oocyte segmentation with the high accuracy guaranteeing their usage as the predefined networks in other tasks.

## Background

Infertility is a wide medical and social problem. The World Health Organization (WHO) defines infertility as a failure to achieve clinical pregnancy after 12 months or more of regular (3-4 times per week) unprotected sexual intercourse [1]. Infertility is considered a disease requiring regular medical care and it constitutes a major problem not only for a given individual, but also for all society. 10–18% of reproductive age partners are affected by infertility worldwide. It is estimated that in Poland, 10–15% or approximately 1.2 million couples struggle with the problem of infertility, with 24000 of them requiring specialist treatment. In Poland there are no detailed statistical studies covering this subject [2–4]. Once infertility is diagnosed, the treatment process involves the techniques of ART (Assisted Reproductive Technology). ART is a group of methods aiming at achieving pregnancy, where a single stage or multiple stages occurring during natural conception are omitted or replaced, depending on the diagnosis and causes of infertility [5]. One of the most effective and frequently applied ART methods is intracytoplasmic injection of sperm (ICSI) [6, 7]. The ICSI method, similar to IVF (In Vitro Fertilization) consists of multiple stages i.a. controlled ovarian hyperstimulation, oocyte retrieval from ovarian follicles, in vitro fertilization of mature oocytes under laboratory conditions, embryo culture and their transfer to the uterine cavity. The procedure results in obtaining one to several dozen oocytes. The condition allowing further stages of the procedure to be carried out is the adequate maturity and quality assessment of the oocyte’s morphological structure. The obtained oocytes are found at various stages of their meiotic maturity. Approximately 80% of the collected oocytes are during the stage of metaphase II meiotic division (MII), remaining 20% are oocytes at the stage of metaphase I (MI), prophase I meiotic division (PI), degenerated cells (DEG) and dysmorphic cells (DYS). Due to low capability of embryonic development, oocytes MI and PI are usually rejected in the process of selection or made to undergo in vitro maturation [8, 9]. The degree of oocyte maturity is determined on the basis of presence of first polar body (FPB) and germinal vesicle (GV) [8, 10].

The quality assessment of oocyte is primarily based on its morphological features observed in a light-microscope. Oocyte quality, and at the same time its development potential, is one of the essential factors determining the success of ART [11, 12]. What is taken into account when assessing the morphological structure of the oocytes is the shape and appearance of cytoplasm, zona pellucida (ZP), perivitelline space (PVS) and FPB. These features are important in terms of a successful fertilization, embryo development and achieving pregnancy and their description and assessment is subjective and depending on the experience and knowledge of the clinical embryologist. One of the biggest problems during oocyte selection is the fact that even a normal looking oocyte can be a carrier of aneuploidy, therefore the research for new methods to simplify the selection oocytes with the highest development potential is in progress [13]. Computer image analysis and the use of artificial intelligence algorithms can be used to solve the problem of optimal selection of oocytes and embryos. Methods of frame-by-frame analysis of embryo culture are commonly used, in which embryo pictures are taken at appropriate time intervals (time-lapse). Basing on the changes found in the appearance of embryos on particular culture days, clinical embryologists are able to assess the development potential. Another research also relates to the appearance of oocytes. For instance, Cavalera et al. [14] combine time-lapse analysis with image anemometry and with use of artificial neural network to determine the movement of cytoplasm in maturing mouse oocytes, thus determining also their development potential with 91.03$\%$ accuracy. Research studies are also underway to develop a method for detecting embryos in the image. For this purpose - a circle detection algorithm based on a modification of Hough transform with Particle Swarm Optimization. Embryo pictures taken directly after carrying out the oocyte fertilization procedure have been tested [15]. Automatic circle detection has been applied to analyze the images of day three embryos. The method has been applied for automatic detection of blastomers [16]. Raudonis et al. [17] propose an automated detection human embryo using a Haar feature-based cascade classifier, the radiating lines and the technique of deep learning obtaining accuracy for embryo detection around 90%. In the paper Singh et al. [18], automatic segmentation of blastomers with the use of ellipsoidal model has been applied, using day one and day two pictures obtained with the use of Hoffman Modulation Contrast. Hierarchical Neural Network in ZP segmentation in human blastocysts was used in subsequent studies [19]. Khan et al. [20] focused on methods of monitoring the developmental stage of the embryo based on the analysis of the image sequence of time-lapse microscopy. The methods made it possible to predict the number of cells with an efficiency of over 90%. Dirvanauskas et al. [21] combined different classifiers to improve the prediction of the development stage of embryos. The best results were achieved after when combining the Convolutional Neural Network (CNN) and Discriminant classifiers. Manna et al. [22] developed the method including a search for patterns in images of oocytes and embryos which could be useful in assessing the development potential. For this purpose, digital images of 269 oocytes and embryos obtained from them have been analyzed, with exclusive focus on the analysis of image covering cytoplasm and blastomers.

The number of oocytes subjected to the procedure depends mainly on the law and patient’s clinical picture. In Poland, a maximum of six oocytes can be fertilized and no more than two embryos can be transferred. The remaining oocytes and obtained embryos are subjected to cryopreservation. An additional question, besides the optimal selection of oocytes for fertilization, is the assessment and classification of development potential of embryos in culture phase and their selection for transfer to the uterine cavity [23, 24]. In some countries the selection of embryos is not possible due to regulations of the law. In Italy it is allowed to create up to three embryos which must be applied during a single transfer procedure into the uterine cavity. Embryo cryopreservation is prohibited except for situations when the implantation is temporarily impossible due to transient health issues [25]. Due to a legal act on embryo protection, the German law prohibits selecting or storing embryos. Transfer of created embryos takes place in the zygote stage on culture day one. Cryopreservation is only allowed in special medical cases [26, 27].

In case of retrieving a big number of oocytes it is important to make the appropriate selection of oocytes to be fertilized. Choosing adequate-quality oocytes constitutes a major medical problem which determines the success of fertilization and further appropriate development of embryos and finally achieving pregnancy.

The subjective nature of morphological assessment of oocytes and embryos is one of the main reasons for seeking non-invasive and— above all—objective methods for assessing quality. Better understanding of the development potential of oocytes and embryos and obtaining new indicators for their selection can increase efficiently the effectiveness of ART treatment [28].

## Results

Bearing in mind state-of-art deep learning models for semantic image segmentation it was decided to exam the major architectures of deep neural networks such as:

- DeepLab v3+ convolutional neural networks
- Fully convolutional neural networks
- SegNet convolutional neural networks
- U-Net convolutional neural networks

Transfer learning technique was adopted due to the small number of learning patterns. In the case of DeepLab v3+ models base networks were specified as ResNet-18, ResNet-50, Xception, or Inception-ResNet-v2. Fully and SegNet convolutional models were initialized using VGG-16 and VGG-19 pretrained networks. U-Net models were used for comparison purposes to verify the case in which a predefined network is not given. Therefore, their convolution layer weights were formed applying the weight initialization method.

One of the problems to be solved during development of the deep neural network for semantic oocyte segmentation is to find the best structure of the neural model, as well as the best parameters of its training process. This task was carried out by using the systematic search procedure. In this way different configurations of the network and training process were examined. For instance, DeepLab v3+ models were modified by changing network parameters, as follows:

- The input image size was chosen from three variants: $\underline{300}$ x $\underline{300}$px, 400 x 400px, 561 x 561px;
- Downsampling factor was set to $\underline{8}$ or 16;

In addition, in the case of fully convolutional models, upsample factor was chosen as 8, 16 or 32, in SegNet models filter size was set to [3 7] or [5 13], whereas in U-Net models encoder depth and number of output channels for first encoder were set to default values.

The stochastic gradient descent with momentum update (Eq. 5) was selected to train neural models. The final result of the training process strongly depends on the values of the behavioural parameters of the training algorithm. Therefore, several variants were examined:

- Momentum coefficient $\gamma$ was equal to 0.8, 0.85, $\underline{0.9}$, 0.95;
- Maximum number of epochs *N* was set to 50, 100, ..., $\underline{500}$, 1000;
- L2 regularization parameter $\kappa$ was set to 1E$-4$ or $\underline{1\hbox {E}-3}$;
- Learn rate drop factor was set to $\underline{0.95}$ or 0.99;
- Normalization weight factor *K* was equal to 25E$+3$, 35E$+3$, $\underline{45\hbox {E}+3}$, 55E$+3$;

The values of other parameters of the algorithm were set as follows: initial learn rate $\alpha$ = 1E$-2$, learn rate drop period = 5, verbose frequency = 8, validation frequency = 10, learn rate schedule was set to ’piecewise’ and shuffling option was set to ’every-epoch’. The underline text indicates values of the behavioural parameters of the learning algorithm for which the best deep neural model has been created in the task of semantic oocyte segmentation.

The whole data set was divided into three separate subsets: T - training data (80%), V - validation data (5%) and TT - test data (15%). Augmentation on the fly technique was applied in order to prevent over-fitting effect. The following image data augmentation operations were used: random rotation, reflection around the X or Y axis, as well as horizontal and vertical translation.

The outcomes of deep learning trials for each deep neural model are shown in Table 1 for training phase and Table 2 for test phase respectively. The orders of the results in the tables are sorted according to weighted intersection over union evaluation metric (wIoU) calculated for test patterns (TT).

**Table 1:** Final results of the experiment of selecting the optimal deep neural network architecture and the values of the training process parameters (training phase)

No.	DNN	Acc		Loss	
		T [%]	V [%]	T	V
1	DeepLab-v3-ResNet-18 (15)	85	79	0.14	0.33
2	DeepLab-v3-ResNet-50 (7)	85	80	0.13	0.40
3	DeepLab-v3-Inception-... (10)	86	80	0.19	0.35
.	.	.	.	.	.
7	DeepLab-v3-Xception (8)	81	80	0.16	0.34
.	.	.	.	.	.
56	fcnLayers (8)	74	77	0.39	0.36
57	SegNetLayers (4)	72	72	0.64	0.77
.	.	.	.	.	.
71	SegNetLayers (7)	50	69	1.82	0.79

**Table 2:** Final results of the experiment of selecting the optimal deep neural network architecture and the values of the training process parameters (test phase)

No.	DNN	wIoU	gAcc				mAcc	mIoU	mBFS
			Avg	Min	Max	Std			
1	DeepLab-v3-ResNet-18 (15)	0.897	0.93	0.79	0.97	0.03	0.74	0.62	0.80
2	DeepLab-v3-ResNet-50 (7)	0.891	0.93	0.74	0.97	0.03	0.75	0.63	0.79
3	DeepLab-v3-Inception-... (10)	0.891	0.93	0.72	0.97	0.04	0.72	0.54	0.79
.	.	.	.	.	.	.	.	.	.
7	DeepLab-v3-Xception (8)	0.883	0.92	0.74	0.97	0.04	0.73	0.56	0.77
.	.	.	.	.	.	.	.	.	.
56	fcnLayers (8)	0.825	0.88	0.66	0.96	0.05	0.64	0.52	0.61
57	SegNetLayers (4)	0.825	0.88	0, 65	0.96	0.06	0.49	0.36	0.66
.	.	.	.	.	.	.	.	.	.
71	SegNetLayers (7)	0.749	0.82	0.58	0.95	0.08	0.32	0.22	0.50

The best score was obtained for the 15th variant of DeepLab-v3-ResNet-18, when the training accuracy (Acc) reached about 85% for training patterns (T) and 79% for validation ones (V). As it was mentioned above, the best configuration of the network structure and its training options and parameters were marked by the underlined text in the previous subsection. Moreover, the smallest value of the categorical cross-entropy loss (Loss=0.33) could be achieved for such a structure of the network, training options and values of parameters. More importantly, it was observed that the given training results allowed to get the very high values of other semantic segmentation quality metrics such as the average boundary F1 contour matching score, as well as ratio of correctly classified pixels to total pixels, regardless of class (gAcc).

**Fig. 1:**
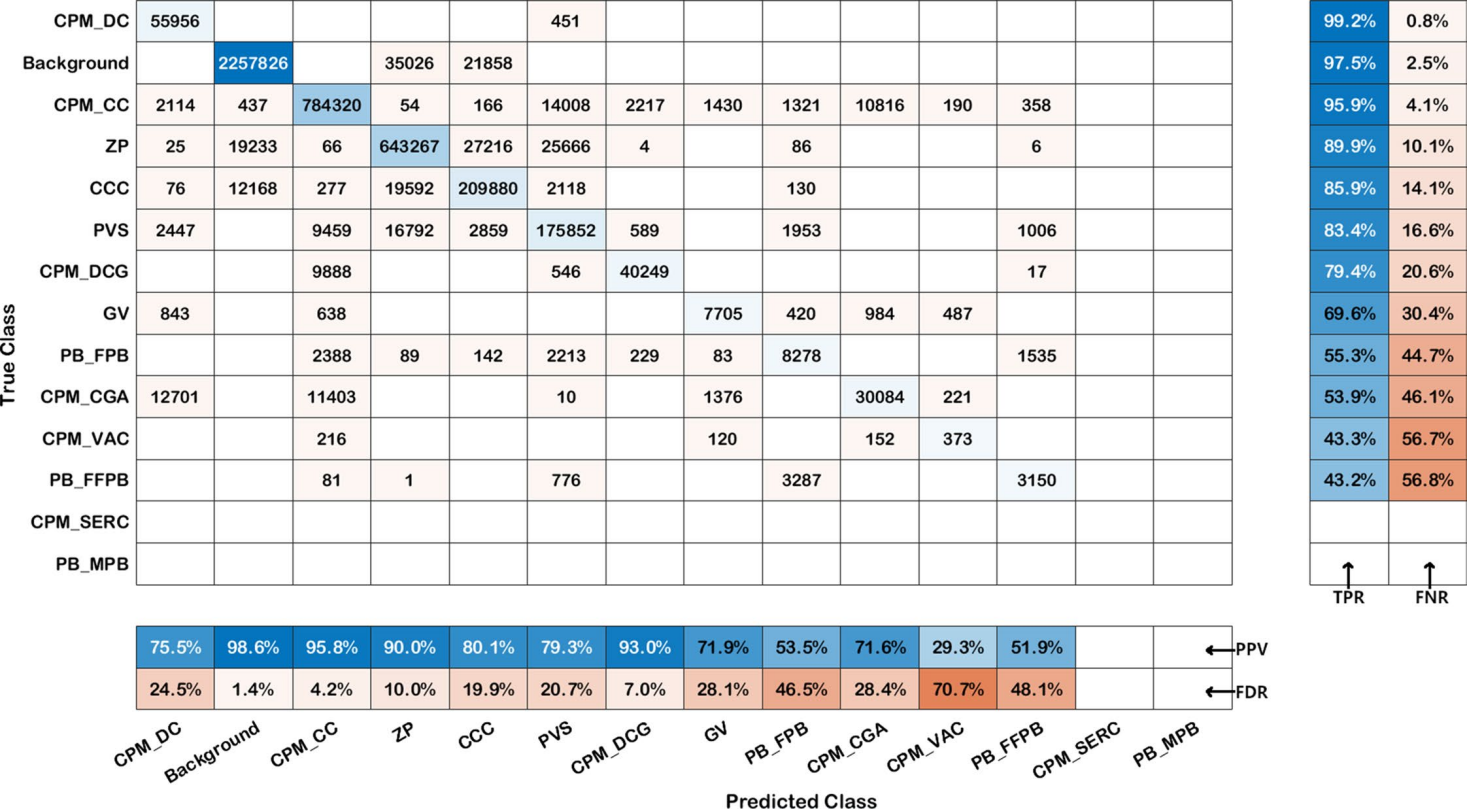
Confusion matrix calculated for DeepLab-v3-ResNet-18 (15) model on test data set (TT)

The more detailed analysis was needed to determine the performance of the DeepLab-v3-ResNet-18 (15) model as an automatic tool for semantic oocyte segmentation. For this reason, the confusion matrix was calculated and charted in Fig. 1. In this way, it was possible to investigate the accuracy of the model taking into account pixel-level classification results for all images. Diagonal and off-diagonal cells of the chart correspond to correctly and incorrectly classified pixels, respectively. The table of confusion was sorted according to the true positive rate.

Pixels belonging to areas such as CPM_DC, CPM_CC, ZP, CCC, PVS and CPM_DCG were segmented without significant mistakes as confirmed by high values of the true positive rate (from 79.4 to 99.2$\%$) and small values of the false discovery rate (from 1.4 to 24.5$\%$). Equally good true positive and false discovery rates were achieved for GV area. Interestingly, CPM_CGA area was identified ambiguously with high value of the positive predictive value (71.6$\%$) and the false negative rate (46.1$\%$). Not very good segmentation results were obtained for CPM_VAC and PB_FFPB areas for which the probability of pixel detection is less than 50$\%$. The accuracy of the segmentation of CPM_SERC and PB_MPB areas was not possible to investigate because of the usage of all images including these pixels in the training stage.

The similar accuracy of semantic oocyte segmentation was observed in training and test phases for other DeepLab v3+ convolutional neural structures which have been created basing on ResNet-50 and Incpetion-ResNet-v2 predefined networks. The values of quality metrics such as gAcc, mAcc, mIoU and mBFS were very close to those in the best solution. For this reason, the additional analysis was needed. The attention was paid to the accuracy of the model corresponding to each segmented area. Table 3 includes outcomes of the accuracy comparison of selected deep neural models in the segmentation task. As one can see, it is not easy to select the best neural model for semantic segmentation of any areas. However, it is possible to answer the question what is the most relevant model for specified area. For instance, it can be stated that the best deep neural model for classification of pixels belonging to CPM_CC is DeepLab-v3-ResNet-18 (15), to CPM_DCG is DeepLab-v3-Incpetion-ResNet-v2 (10), ..., to ZP is DeepLab-v3-ResNet-50 (7) and so on.

**Table 3:** Comparison of the accuracy of selected deep neural models in the segmentation task

DNN	DeepLab-v3-ResNet-18 (15)				DeepLab-v3-ResNet-50 (7)				DeepLab-v3-Inception-.			
Area name	DSC	Acc	IoU	mBFS	DSC	Acc	IoU	mBFS	DSC	Acc	IoU	mBFS
CPM_CC	0.92	0.96	0.92	0.89	0.85	0.94	0.89	0.86	0.88	0.94	0.91	0.89
CPM_DCG	0.46	0.79	0.75	0.64	0.46	0.70	0.53	0.63	0.29	0.85	0.61	0.64
CPM_CGA	0.35	0.54	0.44	0.27	0.28	0.76	0.55	0.43	0.39	0.85	0.60	0.20
CPM_SERC	$-$	$-$	$-$	$-$	$-$	$-$	$-$	$-$	0.10	0.11	0.09	0.56
CPM_VAC	0.16	0.43	0.21	0.96	0.20	0.11	0.11	0.56	0.03	0.08	0.07	0.13
CPM_DC	0.49	0.99	0.75	0.84	0.58	0.95	0.93	0.80	0.49	0.99	0.89	0.85
PB_FPB	0.43	0.55	0.37	0.64	0.56	0.62	0.54	0.67	0.57	0.66	0.54	0.74
PB_MPB	$-$	$-$	$-$	$-$	$-$	$-$	$-$	$-$	0.00	$-$	0.00	$-$
PB_FFPB	0.28	0.43	0.31	0.61	0.27	0.62	0.37	0.67	0.32	0.50	0.40	0.62
PVS	0.79	0.83	0.69	0.93	0.76	0.81	0.70	0.90	0.78	0.86	0.70	0.90
ZP	0.88	0.90	0.82	0.82	0.87	0.92	0.82	0.78	0.84	0.90	0.81	0.76
CCC	0.68	0.86	0.71	0.67	0.70	0.87	0.73	0.65	0.73	0.89	0.75	0.64
GV	0.54	0.70	0.55	0.33	0.46	0.81	0.47	0.53	0.22	0.86	0.33	0.66
Background	0.98	0.98	0.96	0.93	0.98	0.97	0.96	0.92	0.98	0.97	0.96	0.91

Deep learning experiments were carried out employing the personal computer station with Intel®Core™i7-3930K CPU @ 3.20 GHz, 64 GB RAM, 512 GB SSD, 2 TB HDD, NVIDIA™RTX 2080 equipped with 8 GB RAM.

## Discussion

For deeper assessment, it is essential to analyse directly segmentation results obtained for the best and worst deep neural networks. Some examples of segmentation results achieved for test patterns are shown in Table 4. The left part of the table (cells a, d) includes two images of oocytes classified as MII (collected from patient No. 133 and 179, respectively) which have been segmented by clinical embryologist. Whereas, the right part of the table contains a few segmented images of oocytes obtained by means of deep neural networks (cells b, c, e, f). In this part of the table there are included graphical visualisations of differences for both oocyte segmentation methods (human and automatic). The first result of automatic segmentation represents one of the best case obtained by using DeepLab-v3-ResNet-18 (15). Comparing segmentation made by a specialist and segmentation obtained with a deep network it is very hard to observe any differences directly in segmented images. These are only noticeable when we display the diff area of human and automatic segmentation results (the last column of the table). For the first deep network (b) it can be seen that the white and grey pixels cover a very small area of the black image. This looks similar at first sight to the second network (c). However, the diff area exposes discrepancies corresponding to differences between manually and automatically segmented areas, especially in cases such as first polar body FB_FPB, clear cytoplasm CPM_CC, zona pellucida ZP and cumuluse/corona cells CCC. This observation was confirmed for other cases. The least accurate segmentation results were obtained for SegNetLayers network. Images presented in figures (e) and (f) are used to visualise the differences between the deep oocyte segmentation with and without predefined networks. As one can observe, deep neural model created from scratch without predefined network could not guarantee correct results, it means that even straight and easily segementable areas of pixels were portioned into ragged and distorted parts.

**Table 4:**
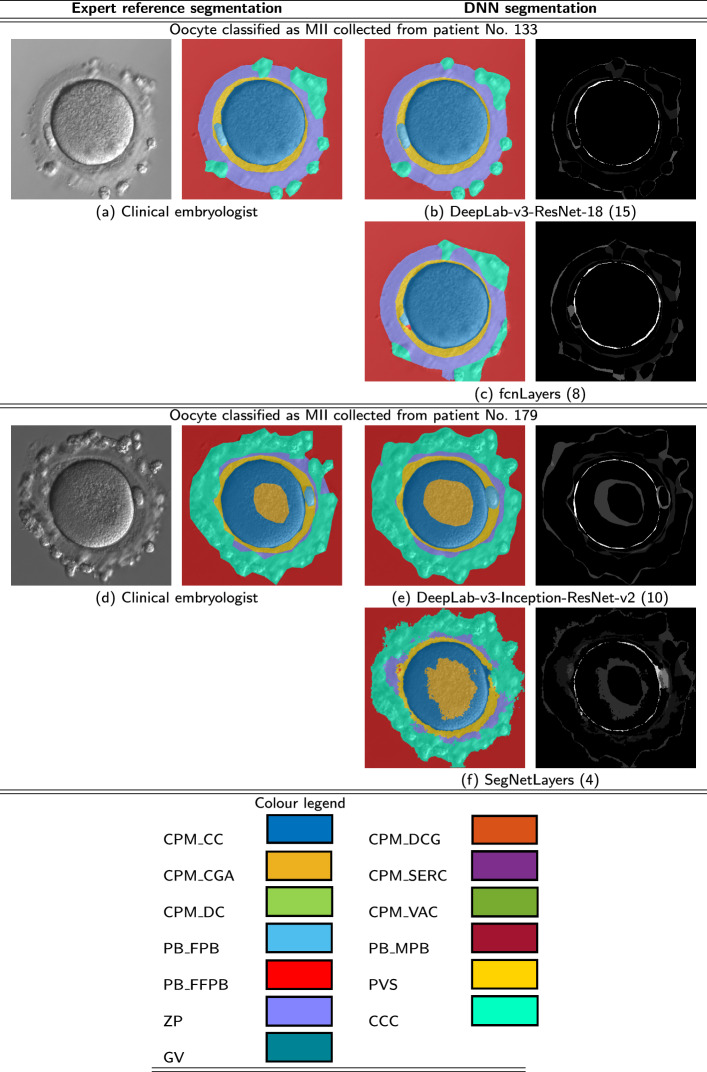
Visual comparison of semantic segmentation results for selected deep models

To understand better the significance of the obtained results the next part of analyses was done taking into account the embryologist’s perspective. Table 5 includes a graphical visualization of segmentation errors. The first column presents the pictures of oocytes. The second column presents manual segmentation carried out by clinical embryologist. The third and fourth column present the result of automatic segmentation and the differences between manual and automatic segmentation.

**Table 5:** Selected results of semantic oocyte segmentation obtained for DeepLab-v3-ResNet-18 (15) model on test data set (TT)

No.	Oocyte image	Expert reference segmentation	DNN segmentation	Differences between expert and DNN segmentation outcomes
1 (PI, patient No. 52)				
2 (MII, patient No. 64)				
3 (PI, patient No. 30)				
4 (MII, patient No. 169)				

It should be emphasized that the problem of oocyte segmentation is a multi-state problem. Basic areas of the oocyte occurring at each developmental stage such as ZP, PVS, CPM_CC and CCC are correctly classified (89.9, 83.4, 95.9 and 85.9%). The obtained results indicate the correct recognition of the area of interest and give a very good prognosis for future works related to the classification of oocytes to specific development stages.

**Fig. 2:**
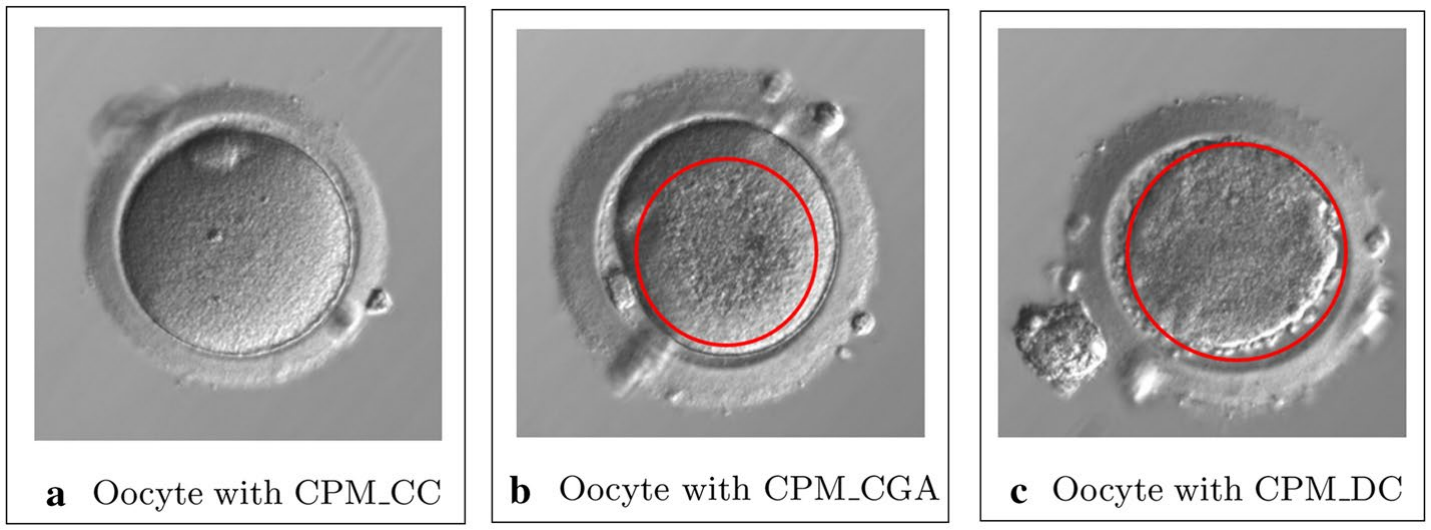
Example of human oocyte images with CPM_CC, CPM_CGA and CPM_DC areas

Globally, the efficiency of segmentation for selected networks is high, nevertheless there are areas where the recognition efficiency is not very good. Having performed the confusion matrix based analysis, one can observe that the error rate for the CPM_CGA areas is higher. This area has been classified improperly with CPM_CC, CPM_DC, CPM_VAC and GV areas, with the largest share in the cumulative error belonging to CPM_CC and CPM_DC areas, which account for 43.2% out of 46.1% of errors. Figure 2 presents 3 images of cells with CPM_CC, CPM_CGA and CPM_DC area. The first figure (2a) presents a cell with pure cytoplasm area. Pure cytoplasm is smooth and bright, whereas the CPM_CGA area presented in the second figure (2b) is darker and has a granular structure. This area is visually similar to area shown in figure three (2c) showing CPM_DC area, while CPM_CGA area occurs only on one fragment of cytoplasm but CMP_DC area covers entire cytoplasm.

Wrong classification of CPM_CGA area as CPM_CC area is not critical for medical reasons, CPM_CGA areas may occur in various sizes and most often centrally located in cytoplasm area. Errors in detecting that area may be a result of mistakes in preparing the training examples. The first example in Table 5 (patient No. 52) shows that the part of region marked by embryologist as CPM_CGA, DNN marked like CPM_DC. The errors could be caused by the locally similar structure of the cytoplasm in both cases, or the darkening of this region due to the presence of CCC.

**Fig. 3:**
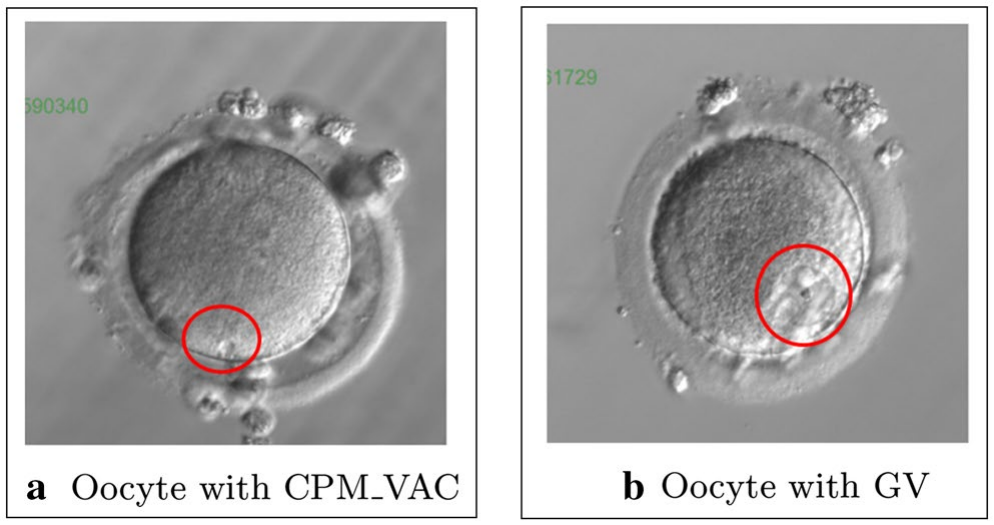
Example of human oocyte images with CPM_VAC and GV areas

CPM_VAC is an area with error rate of 56.7$\%$. This error is mainly related to the failure to recognize small vacuoles (Table 5, patient No. 64) in the cytoplasm or incorrect segmentation of CPM_VAC in the GV structure. Figure 3a presents an oocyte with vacuole, Figure 3b presents an oocyte in PI class with GV.

The vacuole interior background is visually similar to the GV area. This similarity may be the reason for the segmentation error that occurs. The GV structure occurs in and is typical for immature oocytes at PI stage. The lack of vacuole segmentation is related to small areas. Errors generated during segmentation can be related to a small learning set. The test set included four images with vacuoles. On two images the segmentation was correct.

The GV area has been correctly classified in 69.6$\%$. The area was sometimes marked as CPM_CC, CPM_ CGA, CPM_VAC, CPM_DC and PB_FPB. Incorrect segmentation in the cytoplasm area of the oocyte, may be caused by the unclear boundary between the GV region and cytoplasm. An example of mistakes in these region is shown in Table 5, patient No. 30.

Another analysed areas were PB_FPB and PB_FFPB. True positive rate for these regions are 55.3 and 43.2$\%$ respectively. PB_FPB is to the largest rates segmented wrongly as CMP_CC and PVS due to the location of PB_FPB in the cell. Moreover, there are also errors in segmentation concerning images where the PB_FPB is hardly visible due to the presence of CCC, or hidden under the cytoplasm. There is also a problem connected with correct distinguishing between both areas. The example of double segmentation of first polar body is presented in Table 5, patient No. 169. It is planned to unify the PB_FPB and PB_FFPB areas and identify as one area of interest in future research.

It should be noted that the research presented in this paper concerns the segmentation of oocytes in MII, MI, PI, DYS and DEG classes. In this study, the segmentations task concerned 13 different areas, which makes the undertaking very complicated. To the best of the authors’ knowledge, this is the first such a comprehensive study. Other researchers chose to focus on three or four areas. Zhao et al. [29] perform segmentation of day-1 embryos focusing on cytoplasm, ZP and pronuclei. Kheradmand et al. [30] present the segmentation of ICM, TE, cavity and ZP human blastocyst structures. Firuzinia et al. [31] perform segmentation only on mature MII oocytes (Ooplasm, ZP and PVS). In the hereby paper the CPM cytoplasm is divided into sub-areas (CPM_CC, CPM_CGA, CPM_DC, CPM_DGA) and additional structures (CPM_VAC, CPM_SERC, GV). In addition to PVS and ZP areas, the images of oocytes show other important areas such as PB and its sub-areas PB_FPB, PB_MPB and PB_FFPB as well as an additional area of CCC. Due to large disproportions in the number of analyzed areas (3-4 areas vs. 13 areas) it is very hard to submit a direct comparison. In order to obtain the approximate comparison of results, ten images of MII oocytes containing 5 main regions of interest (CPM_CC, PVS, ZP, PB_FPB and CCC) have been selected from the test set. The results of segmentation obtained for these images are presented in Table 6. Moreover, Table 7 presents selected results. Although the network has been designed to recognize 13 areas, it can be seen that the results are comparable to 3-areas segmentation task.

It should be noted that the hereby paper is a part of a project aimed at classification and optimal selection of oocytes and embryos for the IVF procedure. Therefore selecting those many structures has been essential. The ongoing research focuses on the tasks such oocyte classification and the study of impact of the presence of specific structures and their features in correlation with treatment outcomes.

**Table 6:** Comparison of the accuracy with other results presented in literature

		DSC [%]	PPV [%]	TPR [%]
CPM_CC	Proposed method	$98.49 \pm 0.78$	$98.38 \pm 1.68$	$98.61 \pm 0.56$
Ooplasm	Firuzinia et al. [31]	$98.84 \pm 0.23$	$98.72 \pm 0.61$	$98.97 \pm 0.40$
PVS	Proposed method	$85.01 \pm 4.82$	$83.26 \pm 6.41$	$87.06 \pm 4.70$
	Firuzinia et al. [31]	$89.99 \pm 2.86$	$89.81 \pm 2.12$	$90.17 \pm 2.10$
ZP	Proposed method	$92.35 \pm 2.31$	$91.27 \pm 5.00$	$93.70 \pm 2.77$
	Firuzinia et al. [31]	$93.45 \pm 0.78$	$92.82\pm 0.52$	$94.08 \pm 0.68$
	Kheradmand et al. [30]	-	80.3	80.8

**Table 7:** Selected results of semantic segmentation MII oocytes with 5 regions of interest obtained for DeepLab-v3-ResNet-18 (15) model on test data set (TT)

No.	Oocyte image	Expert reference segmentation	DNNsegmentation	Results DSC [%]	
MII P39				CPM_CC	99.04
				PB_FPB	93.28
				PVS	90.85
				ZP	95.52
				CCC	44.47
MII P66				CPM_CC	98.84
				PB_FPB	85.97
				PVS	86.21
				ZP	93.45
				CCC	69.30
MII P124				CPM_CC	98.68
				PB_FPB	80.57
				PVS	88.32
				ZP	94.68
				CCC	-

Finally, it was decided to study the complexity of selected deep models, as well. The most important measures such as computational complexity metric (CCM), total learnables, training and inference time were taken into consideration. According to Table 8, it can be noticed that the DeepLab-v3-ResNet-18 architecture, due to the lowest complexity, has faster inference and training speed. Moreover, the inference speed of this type of the model is higher than in the method proposed by Firuzinia et al. [31]. DeepLab-v3-ResNet-50 and DeepLab-v3-Inception-ResNet-v2 models need much more computing resources. However, the inference time of all selected models is definitely acceptable from a practical point of view. Hence, it may be concluded that, analysing an oocyte image can be done by means of such models in real-time, even on a personal laptop computer.

**Table 8:** Comparison of the selected deep neural networks in terms of computational complexity, the number of learnable parameters and training/inference speed

Deep model	CCM	Total learnables	Training-Time	Inference-Time
	[GFLOPs]	[millions]	[s/epoch]	[ms]
DeepLab...ResNet-18 (15)	3.637	$\sim$ 17.5	35	36
DeepLab...ResNet-50 (7)	8.2164	$\sim$ 43	376	105
DeepLab...Incpetion...v2 (10)	22.227	$\sim$ 71	623	179

## Conclusion

This paper is focused on a method of semantic segmentation of human oocytes by means of deep neural networks. The performance comparison of different types of convolutional neural networks for semantic oocyte segmentation was carried out. The merits and limitations of the selected deep neural networks were discussed. As a result, it has been proved that the proposed approach can be used to create deep neural models for semantic oocyte segmentation with high accuracy. In effect, such models can be employed as the predefined networks in other tasks. To the best of the authors’ knowledge, this research is the largest study to date in the context of semantic segmentation of human oocytes using deep neural networks. The data set of 334 pictures of oocytes has been used in this paper (segmented by a clinical embryologist). It should be emphasised that, 13 areas of interest typical for cells at various stages of their development have been identified. The main purpose of the paper was to recognize deep neural networks optimal for the task of segmenting human oocytes. This paper involves the examination of 71 deep neural models and it has been found by ${\varvec{{wIoU}}}$ that the best global results were achieved using the DeepLab-v3-ResNet-18 model. Computational complexity and comparative analysis for selected neural networks were performed.

Due to a relatively small number of training examples and significant differences in numbers of pixels representing particular areas of the cell structure, some areas were prone to bigger prediction error. What is a very big advantage of the proposed methodology is the fact that thanks to automatic segmentation it will be possible to analyse automatically particular areas and estimate their typical statistical features, it will be possible to analyse absolute measures such as the size of the surface of a specific area, as well as relative measures. In the next stages of the study, the authors will examine this problem hypothesizing that these features might be a carrier of diagnostic information.

This study is a part of a wider research on the development of an optimal selection system for oocytes to be subjected to in vitro fertilization. In the next step the system will be expanded with optimal embryo selection module. Classification of oocytes is a complex task and the authors have assumed that better classification results will be achieved with the use of deep neural networks which recognize and properly segment the areas visible in the image of the oocytes.

The results presented in the hereby paper can be employed to build an advisory system used to support the work of a clinical embryologist, as well as to develop a training/educational system which provides the possibility to verify the correct determination/marking of particular structures. It has to be emphasized that the proposed method is classified to the group of soft computing approaches. The well-known and often practised validation technique was used to assess how the model will generalize to an independent data set. However, this does not guarantee the proposed method will work correctly on all new data.

## Methods

**Fig. 4:**
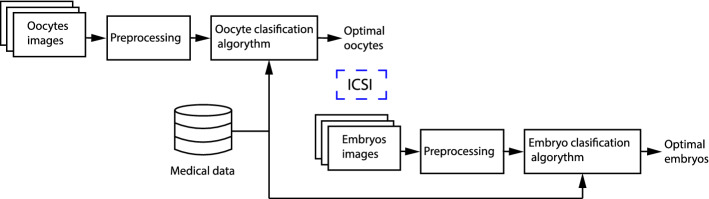
Methodology of selecting optimal oocytes and embryos

The suggested methodology for optimal choice of oocytes and embryos is schematically presented in Fig. 4. In compliance with the presented methodology, oocyte pictures are taken directly after the denudation process. The pictures are pre-edited (scaled, centred, resampled). Properly prepared digital images and remaining medical data collected during treatment and standard diagnostic tests constitute the input of the algorithm for optimal selection of oocytes to be successively subjected to ICSI procedure.

The obtained embryos are subjected to observation throughout the next culture days (1-6) and their appearance is registered. The sequence of embryo pictures along with medical data is evaluated, similarly to oocytes. The algorithm indicates optimal embryos which reveal the best development potential.

In compliance with the methodology, the first stage is the optimal selection of oocytes. The hereby work focuses on the use of deep learning methods. Approaches to automatic classification of oocytes and embryos involving this kind of methods are known in the literature [14, 22, 32, 33]. This approach consists of providing a picture to the network which then classifies and assigns the picture to a given quality group. One can assume that the network is not taught to recognize particular morphological structures.

**Fig. 5:**
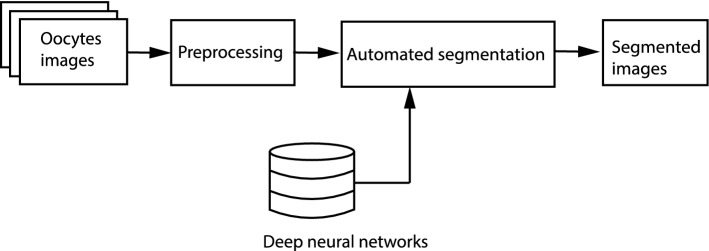
Method of segmentation of oocyte images

On the contrary to the presented works, it has been assumed that the classifying network will be pre-trained in terms of recognition and segmentation of human embryos. It has been hypothesized that training the classification network will be more effective if the network “understands” the content of the picture. What is an additional advantage of such approach is the possibility to use segmented pictures to determine various measures and statistical features of the analyzed areas. The analysis of particular areas will be relatively easier if e.g. the shape, surface area of the zones of interest, etc. are known. Figure 5 is a schematic presentation of the methodology of conduct in automatic segmentation of oocytes.

### Oocyte–morphological structures in microscopic image

**Fig. 6:**
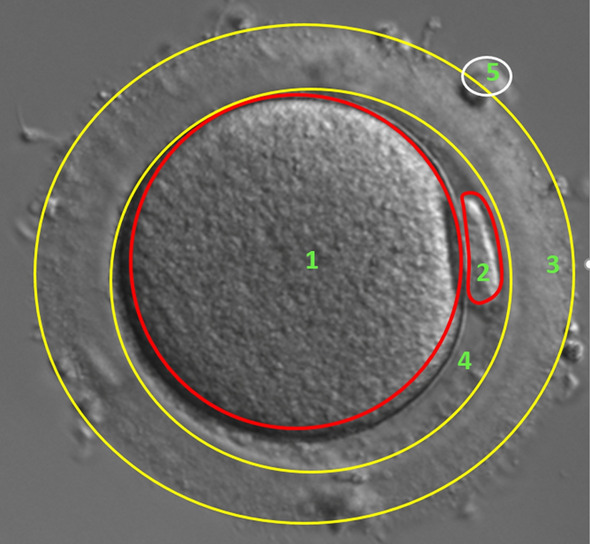
Image of oocyte in stage MII (1—Cytoplasm (CPM), 2—First polar body (FPB), 3—Zona pellucida (ZP); 4— Perivitelline space (PVS); 5—Cumulus/corona cells (CCC))

Figure 6 presents an image of an oocyte in MII class. The total diameter of a mature oocyte is approximately 150 $\mu m$. A mature oocyte consists of oolemma-surrounded cytoplasm (CPM) (1) with a diameter of about $110-115$ $\mu m$, first polar body (2), $15-20$ $\mu m$ wide pellucid zone (3), perivitelline space (4), the remains of granulosa cells Cumulus/corona cells (CCC) (5) are usually visible in the pictures of oocytes [24, 34].

The assessment of maturity and morphological structure quality is performed after the process of denudation, that is the purification of oocytes from the surrounding cumulus, which is a cumulus-oocyte complex (COC) [10]. Oocytes occur at different stages of their development (MII, MI, PI, DEG, DYS) and contain different morphological structures. Table 9 presents the images of 13 morphological identified structures. The occurrence of specific structures and the assessment of their morphology is the basis for oocyte qualification and assessment of its development potential.

**Table 9:** Segments of oocytes

Clear cytoplasm	Diffuse cytoplasmic granularity	Cytoplasmic granular area	Smooth endoplasmic retculum cluster	Dark cytoplasm
CPM_CC	CPM_DCG	CPM_CGA	CPM_SERC	CPM_DC
Vacuoles	First polar body	Multi polar body	Fragmented first polar body	Perivitelline space
CPM_VAC	PB_FPB	PB_MPB	PB_FFPB	PVS
Zona pellucida	Cumulus/corona cells	Germinal vesicle		
ZP	CCC	GV		

Five areas have been distinguished in the cytoplasm group. Pure CPM_CC cytoplasm with a smooth and homogeneous surface. CPM_DCG dispersed granularity cytoplasm, characterized by significant and even granularity in the image. CPM_CGA cytoplasm granularity area in which a distinct, darker granularity zone can be distinguished, with the rest of cytoplasm being smooth. Smooth endoplasmic reticulum cluster CPM_SERC, which has a smooth and oval surface with a clearly visible edge in the cytoplasm area. Dark cytoplasm CPM_DC - an area typical for degenerated cells of a clearly dark color, without visible depth. The last area identified in the cytoplasm are the Vacuoles CPM_VAC, which form clearly visible oval craters. The next cell structure is the PB (polar body). In terms of morphology and quantity, three types of polar body have been identified. The polar body is located between the ZP and the cytoplasm. First Polar Body PB_FPB has a smooth, homogeneous surface, most often it has an ellipsoidal shape. The occurrence of fragmentation in first polar body determines it to be qualified for the area called PB_FFPB. There may be more polar bodies in the oocyte—this is referred to as the Multi polar body PB_MPB.

There is a PVS perivitelline space between the oolemma and the zona pellucida. There may be some granularity in its area. Oocyte is surrounded by zona pellucida. ZP has a porous, homogenous surface. There might be spherical structures and granullity CCC on the ZP surface. The last identified structure is the GV present in oocytes in the PI phase. GV occurs in cytoplasm area, it is a circle with a smooth structure and clear edges, containing a clearly visible spherical nucleus on its surface.

### Data set preparation

Oocytes have been collected from 60 patients (average age of 32 ± 10 years) subjected to ICSI procedure. In total 334 pictures of oocytes have been used, including 236 pictures of oocytes classified as MII, 21 as MI, 48 as PI, 8 as DYS and 23 as DEG. The patients were subjected to hormonal stimulation. The ovarian stimulation protocol was chosen based on the clinical picture. After collection, the COC were incubated for $2-5$ hours in culture medium (SAGE 1-Step™, Origio CooperSurgical Companies) in an incubator at $37^{\circ }$C, $6\%$ $\hbox {CO}_2$. Oocytes were subjected to denudation of granular cells by exposure to 80 IU/ml hyaluronidase (GM501 Hyaluronidase; Gynemed Germany) for 1 minute and mechanically cleaned.

Pictures were taken with use of an inverted light-microscope (Olympus®, IX51/IX70) at x200 magnification, using a camera (Oosight CCD Camera Module) and Oosight®Meta software (Hamilton Thorne, Inc.). The recorded image may contain one or more oocytes and micromanipulation needles, on condition it did not affect the individual shape of each oocyte. The recorded images were pre-edited, which resulted in obtaining 561 x 561 pixels dimensions, saved as .bmp files in greyscale. In order to prepare learning patterns, each image underwent manual segmentation. The segmentation was carried out employing Image Labeler application available as part of MATLAB®R2019b software. With use of the application, each of the 334 images was manually segmented.

**Fig. 7:**
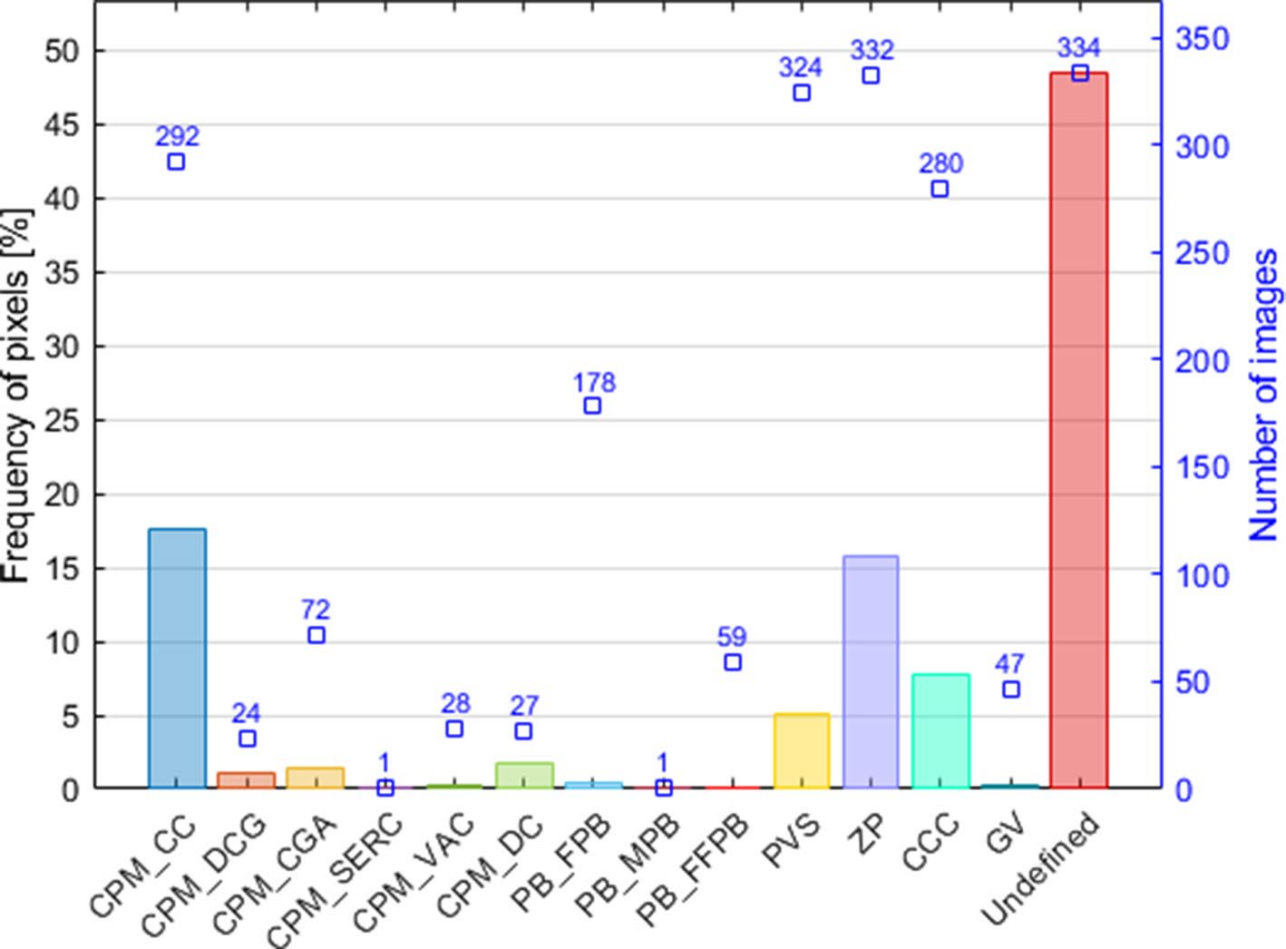
Graphical description of the learning data set

In the next step, the entire data set including manually segmented images was analysed in detail to obtain statistical description of the set of deep learning patterns. The most important parameters of the data set are summarised in Fig. 7. This chart shows values of the frequency of pixels calculated for different areas of the image resulting from morphological structure analysis of the oocyte. This parameter is very important in the context of the automation of segmentation process by means of deep neural networks.

It should be noted here that there is a lot of learning examples in the collected data set containing the group of areas of interest such as CPM_CC, PVS, ZP, CCC as well as undefined pixels (from 280 to 334 images). As one can see, in average undefined pixels cover nearly the half of each image. However, the value of the frequency of pixels for significant areas in this group is relatively high. The next group of areas such as CPM_DCG, CPM_CGA, CPM_VAC, CPM_DC, PB_FPB, PB_FFPB and GV appears on several different images (from 24 to 178 images). The part of this group includes areas where the value of the frequency of pixels is larger then 1$\%$, but it has also areas where this value is smaller or significantly smaller then 1$\%$. The most problematic areas in this case study are CPM_SERC and PB_MPB. There is only one image for each case and, in effect, the value of the frequency of pixels is extremely low.

### Deep semantic oocyte segmentation method

Semantic oocyte segmentation is the task of labelling every pixel in an oocyte image with a pre-defined area category and it must be usually solved when the detailed understanding of such image is required. In other words, the term suggests this is the process of dividing an oocyte image into multiple segments such as cytoplasm, first polar body, zona pellucida, etc. Semantic oocyte segmentation task can be done in automatic manner by means of deep neural networks which have yielded a new generation of image segmentation models with remarkable performance improvements. In this section, the main issues of deep semantic oocyte segmentation method are discussed.

#### Applied deep segmentation models

As one can see in [35], there are several major types of deep neural architectures for image segmentation such as: fully convolutional networks [36, 37], convolutional networks with graphical models i.e. the combination of convolutional neural networks and fully connected conditional random fields [38], encoder-decoder models for general segmentation [39] or for medical image segmentation [40, 41], multi-scale and pyramid network based models [42], dilated convolutional models and DeepLab family [43], and many others. In this paper it was decided to apply and compare four different architectures which are described below.

*Fully Convolutional Network*

**Fig. 8:**
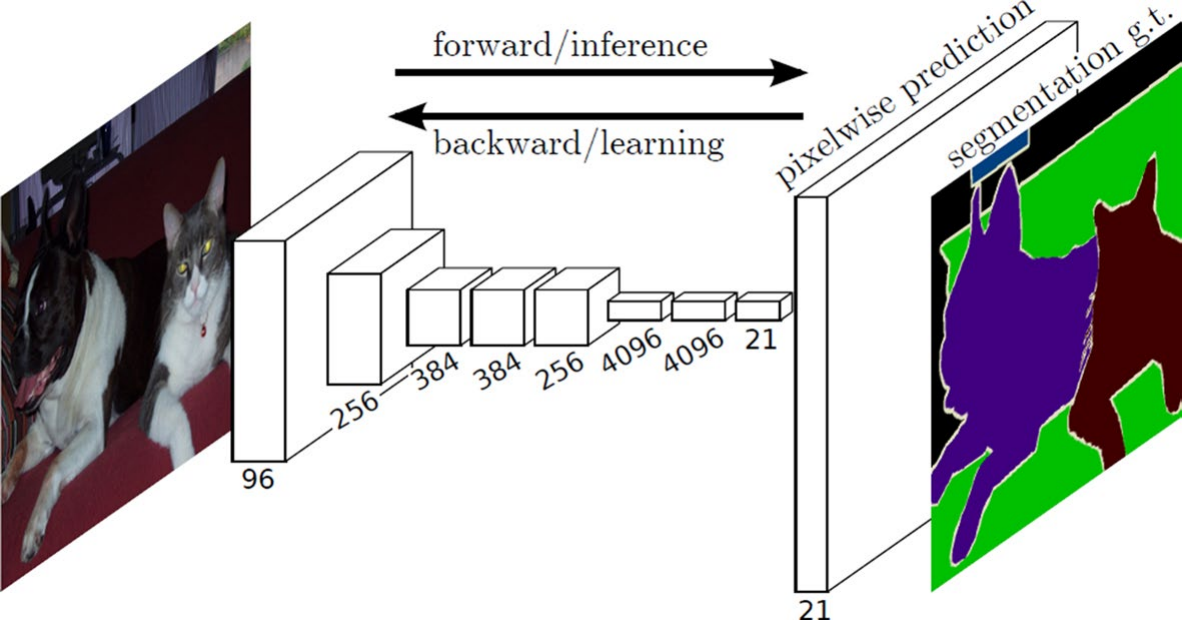
Fully convolutional image segmentation network [36]

A fully convolutional network presented in Fig. 8 includes convolutional and pooling layers. Long et al. [36] modified existing CNN architectures (i.e. VGG16) by replacing all fully-connected layers with the fully-convolutional layers to obtain mapping from pixels to pixels, without extracting the region proposals. Such network takes an image of arbitrary size and produces a segmentation map of the same size.

Moreover, authors [36] proposed the skip connections to combine semantic information from deep, coarse layers and appearance information from shallow, fine layers to produce accurate and detailed segmentations. This structure of a network is considered a milestone in image segmentation.

**Fig. 9:**
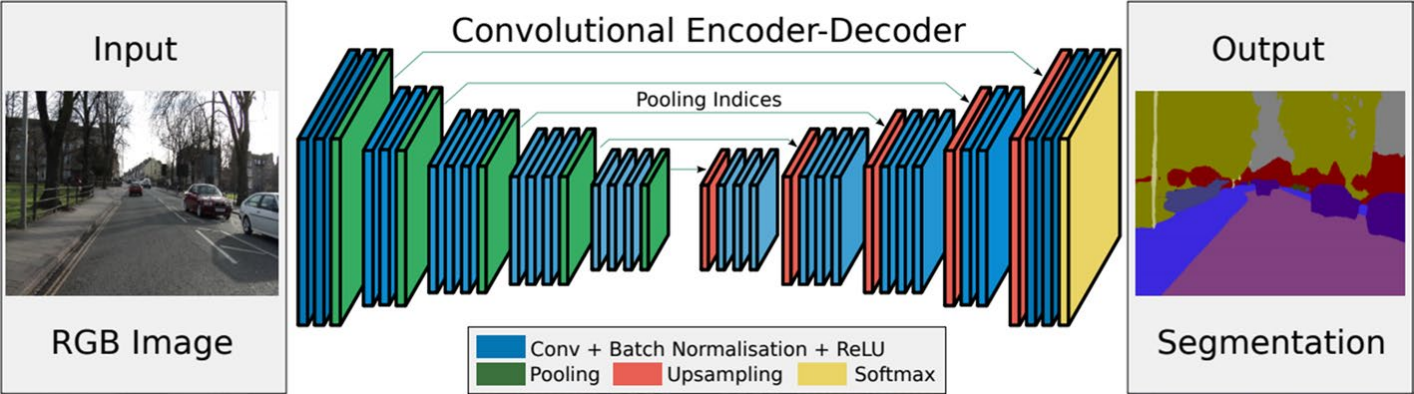
SegNet—fully convolutional network [39]

*SegNet* SegNet was proposed by Badrinarayanan et al. [39] as a convolutional encoder-decoder architecture for semantic pixel-wise segmentation (Fig. 9). In this type of a network, the trainable part of SegNet is composed of an encoder network (similar to the 13 convolutional layers in the VGG16 network), as well as a corresponding decoder network followed by a pixel-wise classification layer.

SegNet is less complex than other competing architectures in the context of the number of trainable parameters. This network is more efficient since it only stores the max-pooling indices of the feature maps and uses them in its decoder network to achieve good performance [39].

*U-Net*

**Fig. 10:**
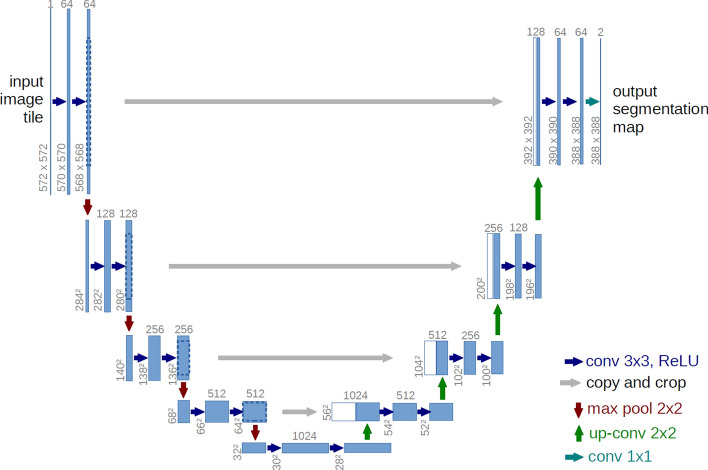
U-net architecture [40]

U-Net (Fig. 10) is inspired by FCNs and encoder-decoder models, and it was initially developed for medical/biomedical image segmentation. Specifically, Ronneberger et al. [40] elaborated this architecture for segmenting biological microscopy images. The structure of the network consists of a contracting path to capture context and a symmetric expanding path that enables precise localization.

*DeepLab3v+*

**Fig. 11:**
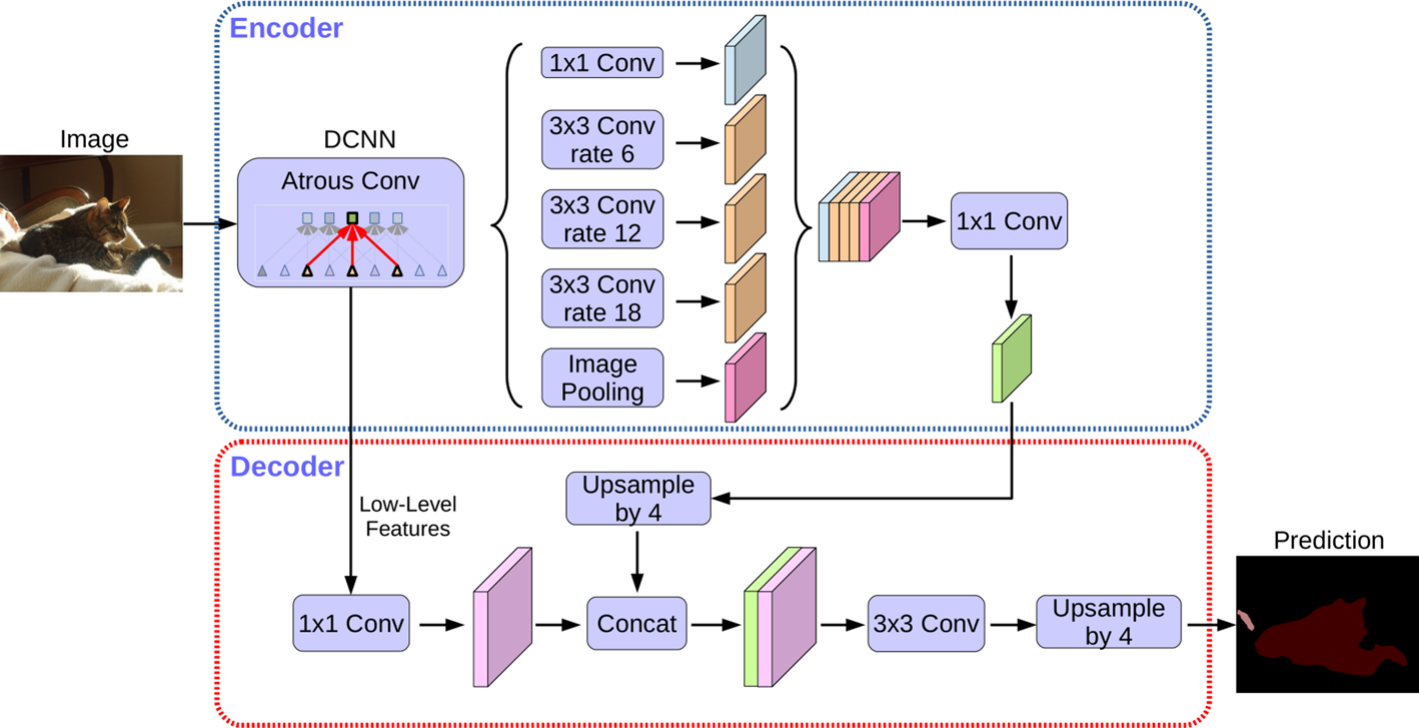
DeepLabv3+ with a encoder-decoder structure [43]

There is a DeepLab family of networks developed by Chen et al. One of the newest models of this type is known as Deeplabv3+ (Fig. 11). This network uses an encoder-decoder architecture, including atrous separable convolution which is composed of a depthwise convolution and pointwise convolution.

#### Deep learning algorithm

Different kinds of learning algorithms can be used for updating the network parameters (weights and biases) in order to minimize the loss function. In this paper, the loss function with the regularization term is formulated as follows:

1 $$\begin{aligned} E_R\left( {\varvec{\Omega }}\right) = E\left( {\varvec{\Omega }} \right) + \lambda \kappa \left( {\varvec{\omega }}\right) \end{aligned}$$

where ${\varvec{\Omega }}$ is the network parameters vector, $\lambda$ is the regularization factor (coefficient) responsible for emphasizing the regularization function which is needed to reduce over-fitting problem. The regularization term is proposed as weight decay and it is given in the form of the formula:

2 $$\begin{aligned} \kappa \left( {\varvec{\omega }}\right) = \frac{1}{2} {\varvec{\omega }}^T{\varvec{\omega }} \end{aligned}$$

where ${\varvec{\omega }}$ is the weight vector.

The main formula component $E\left( {\varvec{\Omega }} \right)$ is the weighted cross-entropy function given by the following equation:

3 $$\begin{aligned} \begin{aligned} E\left( {\varvec{\Omega }} \right) = - \frac{1}{N} \sum _{i=1}^N\sum _{j=1}^C c_j\left[ T_{i,j} \log \left( X_{i,j} \right) + \left( 1 - T_{i,j}\right) \log \left( 1-X_{i,j}\right) \right] \end{aligned} \end{aligned}$$

where *N* is the number of training patterns, *C* is the total number of categories, $X_{i,j}$ is the network response for a given category, $T_{i,j}$ is the target value of that category, $c_{j}$ is the weight of the *j*-th category. This form of the cross-entropy loss is necessary in classification problems with an imbalanced distribution of classes. In this paper, the values of weights $c_j$ are determined using the frequencies of pixels in the image data set. Heuristic rule is applied to compute the values of frequency class weights:

4 $$\begin{aligned} c_j = \log \left[ \frac{K}{f_jf^m_j}\right] \end{aligned}$$

where *K* is the normalization weight factor, $f_j$ is the frequency of occurrence of pixels for the *j*-th area in the whole data set of images, $f^m_j$ is the value of the frequency of occurrence of pixels for an area including the largest number of pixels.

The stochastic gradient descent with momentum rule [44] is applied to find minimum of the loss function (Eq. 1). In this algorithm, values of network parameters are updated, at each iteration in the direction of the negative gradient of the loss, as follows:

5 $$\begin{aligned} {\varvec{\Omega }}_{n+1} = {\varvec{\Omega }}_{n} - \alpha \nabla E_R\left( {\varvec{\Omega }}_{n}\right) + \gamma \left( {\varvec{\Omega }}_{n} -{\varvec{\Omega }}_{n-1} \right) \end{aligned}$$

where *n* is the iteration number, $\alpha$ is the learning rate, $\gamma$ determines the contribution of the previous gradient step to the current iteration.

#### Transfer learning

Transfer learning is one of the machine learning techniques to speed up training and improve the performance of a deep learning model. In this method pre-trained neural models are used as the starting point for further improvements in the context of the new task. Different generally available deep neural models can be used as a network backbone. This study makes use of the following pre-defined networks: ResNet-18 and ResNet-50 [45], Xception [46], Inception-ResNet-v2 [47], VGG-16 and VGG-19 [48].

#### Data augmentation

Data augmentation is a data processing technique used to increase the number of labeled samples, especially when learning from limited data sets, such as those in medical image analysis (in classification and segmentation tasks). This serves to increase the number of training samples by applying a set of transformation to the images (i.e., both the input image and the segmentation map. Using this technique frequently leads to faster convergence, decreasing the chance of over-fitting, and enhancing generalization [35]. Various transformation operators can be applied such as translation, reflection, rotation, warping, scaling, color space shifting, cropping, and projections onto principal components. A survey on image data augmentation for deep learning is given by Shorten and Khoshgoftaar [49].

#### Estimation of deep model accuracy

The quality of semantic segmentation results against the ground truth segmentation can be evaluated using various metrics [50]. In this paper, the following semantic segmentation metrics are taken into account:

- Accuracy (**Acc**) - for each class, accuracy is the ratio of correctly classified pixels to the total number of pixels in that class, according to the ground truth. There are two variants of this measure: $${\varvec{{gAcc}}}$$ is the ratio of correctly classified pixels, regardless of class, to the total number of pixels; $${\varvec{{mAcc}}}$$ is the average $${\varvec{{Acc}}}$$ of all classes in all images.
- True positive rate (**TPR**) - is also known as sensitivity, recall or hit rate and it describes the relation between true positives and all positive elements:
  6 $$\begin{aligned} {\textbf{{TPR}}} = \frac{\text{TP}}{\text{TP+FN}} \end{aligned}$$
  where *TP* is the number of true positives, *FN* is the number of false negatives.
- False negative rate (**FNR**) - or miss rate, it corresponds to the proportion of positive pixels which yield negative test outcomes with the test:
  7 $$\begin{aligned} {\textbf{{FNR}}} = \frac{{\text{FN}}}{{\text{FN}}+{\text{TP}}} = 1 - {\text{TPR}} \end{aligned}$$
- Positive predictive value (**PPV**) - is also known as precision and it represents the relation between true positives and all elements segmented as positive
  8 $$\begin{aligned} {\textbf{{PPV}}} = \frac {\text{TP}}{\text{TP}+\text {FP}} \end{aligned}$$
  *FP* is the number of false positives.
- False discovery rate (**FDR**) - it describes the expected proportion of type I errors.
  9 $$\begin{aligned} {\textbf{{FDR}}} = \frac {\text{FP}} {\text{FP}+\text{TP}} = 1 - \text{PPV} \end{aligned}$$
- Intersection over union (**IoU**) - is also known as the Jaccard similarity coefficient. This metric is used as a statistical accuracy measurement that penalizes false positives. For each class, it is the ratio of correctly classified pixels to the total number of ground truth and predicted pixels in that class
  10 $$\begin{aligned} {\textbf{{IoU}}} = \frac{\text{TP}}{\text{TP}+{\text{FP}}+{\text{FN}}} \end{aligned}$$
  The value of $${\varvec{{IoU}}}$$ for each class is weighted by the number of pixels in that class and marked as $${\varvec{{wIoU}}}$$ to reduce the impact of errors in the small classes on the aggregate quality score. For the aggregate data set $${\varvec{{mIoU}}}$$ is the average $${\varvec{{IoU}}}$$ score of all classes in all images.
- The boundary F1 contour matching score - is used to indicate how well the predicted boundary of each class aligns with the true boundary, and it is used to correlate better with human qualitative assessment than the $${\varvec{{IoU}}}$$ metric. It can be written as follows:
  11 $$\begin{aligned} {\textbf{{BFS}}} = 2 \frac{{\text{PPV}} \cdot {\text
{TPR}}}{\text{PPV} +{\text{TPR}}} \end{aligned}$$
  In this paper, the average BF score of that class over all images or the average BF score of all classes in all images are computed ($${\varvec{{mBFS}}}$$).
- Sørensen-Dice similarity coefficient (**DSC**) - which is a spatial overlap index, measures the overall segmentation accuracy between the manual and automatic segmentations
  12 $$\begin{aligned} {\textbf{{DSC}}} = \frac{2{\text{TP}}}{2{\text{TP}}+{\text{FP}}+{\text{FN}}} = \frac{2{\text{IoU}}}{1+{\text{IoU}}} \end{aligned}$$
  The Dice coefficient is related to the Jaccard coefficient.

## Data Availability

Not applicable

## Abbreviations

ACC: Accuracy
gACC: global Accuracy
mACC: average Accuracy
ART: Assisted Reproductive Technology
BFS: Boundary F1 contour matching score
mBFS: average Boundary F1 contour matching score
CNN: Convolutional Neural Network
CPM: Cytoplasm
CCC: Cumulus/corona cells
CCM: Computational complexity metric
COC: Cumulus-oocyte complex
CPM_CC: Cytoplasm with a smooth and homogeneous surface
CPM_DCG: Dispersed granularity cytoplasm
CPM_CGA: Cytoplasm granularity area
CPM_SERC: Smooth endoplasmic reticulum cluster
CPM_DC: Dark cytoplasm
CPM_VAC: Vacuoles
DEG: Degenerated cells
DSC: Sørensen-Dice similarity coefficient
DYS: Dysmorphic cells
DNN: Deep Neural Networks
FCN: Fully Convolutional Network
FDR: False discovery rate
FNR: False negative rate
FPB: First polar body
GFLOPs: Giga floating point operations per second
GV: Germinal vesicle
ICSI: Intracytoplasmic Sperm Injection
IVF: In Vitro Fertilization
IoU: Intersection over Union
wIoU: weighted Intersection over Union
mIoU: average Intersection over Union
MII: Metaphase II stage oocytes
MI: Metaphase I stage oocytes
PI: Prophase I stage oocytes
PB: Polar body
PB_FPB: First polar bod
PB_FFPB: Fragmented first polar body
PB_MPB: Multi polar body
PVS: Perivitelline space
PPV: Positive predictive value
TPR: True positive rate
TT: Test patterns
T: Training patterns
V: Validation patterns
WHO: World Health Organization
ZP: Zona pellucida
